# Des-γ-Carboxyprothrombin (DCP) and NX-DCP Expressions and Their Relationship with Clinicopathological Features in Hepatocellular Carcinoma

**DOI:** 10.1371/journal.pone.0118452

**Published:** 2015-03-04

**Authors:** Akiko Sumi, Jun Akiba, Sachiko Ogasawara, Masamichi Nakayama, Yoriko Nomura, Makiko Yasumoto, Sakiko Sanada, Osamu Nakashima, Toshi Abe, Hirohisa Yano

**Affiliations:** 1 Department of Pathology, Kurume University School of Medicine, Kurume, Fukuoka, Japan; 2 Department of Radiology, Kurume University School of Medicine, Kurume, Fukuoka, Japan; 3 Department of Clinical Laboratory Medicine, Kurume University Hospital, Kurume, Fukuoka, Japan; University Hospital of Essen, GERMANY

## Abstract

**Aim:**

Des-γ-carboxyprothrombin (DCP) has been used as a tumor marker for hepatocellular carcinoma (HCC). Recently the DCP/NX-DCP ratio, calculated by dividing DCP by NX-DCP, has been reported useful in detecting HCC. The purpose of this study is to clarify the significance of DCP and NX-DCP expression in HCC tissues.

**Methods:**

HCC and non-HCC tissue samples were obtained from 157 patients and were immunohistochemically examined for DCP and NX-DCP expression using anti-DCP antibody and anti-NX-DCP antibody. DCP and NX-DCP expression scores were calculated by multiplying staining intensity grade by percentage of stained area. Serum DCP and NX-DCP levels were determined in 89 patients. We evaluated the relationship between tumor expression, serum level, and pathomorphological findings.

**Results:**

Intrahepatic metastasis (im) was significantly more frequent in cases with high DCP expression than in cases with low DCP expression. High NX-DCP expression was associated with significantly lower histological grade, and less frequent im or portal vein invasion (vp) than low NX-DCP expression. Serum DCP was correlated with DCP expression, but serum NX-DCP was not correlated with NX-DCP expression. DCP-positive (≥40 mAU/L), NX-DCP-positive (≥90 mAU/L), and DCP/NX-DCP ratio-positive (≥1.5) cases were associated with significantly larger tumor size and more frequent vp than negative cases. DCP was rarely expressed, but NX-DCP was frequently expressed in non-cancerous liver tissues. Patients with NX-DCP expression-negative tumors showed a lower survival rate than those with NX-DCP expression-positive tumors (p = 0.04), whereas the survival in serum NX-DCP-positive cases was lower than that of serum negative cases (p = 0.02).

**Conclusions:**

DCP and NX-DCP were produced in HCC tissues, but differed in expression level and biological properties. DCP expression, serum DCP or NX-DCP level, and DCP/NX-DCP ratio were closely related to malignant properties of HCC.

## Introduction

Hepatocellular carcinoma (HCC) is the third most common cause of cancer death in the world. Advances and improvements in the screening and treatment of patients at high risk for HCC have improved the prognosis of early-stage HCC [1]. However, the prognosis of advanced HCC remains extremely poor, even after the induction of the multi-tyrosine kinase inhibitor, Sorafenib.

Des-γ-carboxyprothrombin (DCP), also known as “protein induced by vitamin K absence or antagonist-II” (PIVKA-II), is an abnormal prothrombin that has been widely used as a tumor marker for HCC, and could be predictive of worse tumor behavior and prognosis [2–5]. Furthermore, it could be a preoperative predictor of poor prognosis in patients undergoing living donor liver plantation [5–7]. Amino acid residues differ between normal prothrombin and DCP. DCP has several variants based on the number of glutamic acid (Glu) residues and their positions in the γ-carboxyglutamic (Gla) domain. In patients with HCC, DCP has been identified using MU-3 antibody, which reacts strongly with DCP variants containing few Gla residues. Serum DCP also increases in patients with vitamin K deficiency, such as those taking warfarin, or who have obstructive jaundice. DCP elevated in such conditions was found to contain more Gla residues and was named NX-DCP. NX-DCP can be detected using P-11 or P-16 antibody [8–13]. Recently there are some reports that serum NX-DCP level and the DCP/NX-DCP ratio calculated by dividing serum DCP level by serum NX-DCP level are useful for diagnosis and prognosis of HCC [10, 12, 14, 15]; however, little is known about tissue NX-DCP expression [16], and no large cohort study has been done. The purpose of this study is to clarify the significance of DCP and NX-DCP expression in HCC and non-cancerous tissues, and to analyze the relationship between serum DCP and NX-DCP levels and DCP and NX-DCP expression in HCC tissues.

## Materials and Methods

HCC tissue samples for immunohistochemistry were obtained from 157 patients who underwent surgical resection of single HCC nodules at Kurume University Hospital between 2007 and 2012. Of these cases, 6 patients had been taking warfarin. Non-cancerous liver tissues were available from 148 cases. None of the patients had previously received any treatments, including arterial embolization, chemotherapy, or radiofrequency ablation. Patients consisted of 117 men and 40 women aged from 32 to 84 years (median age 68 years). Eighty-nine cases were hepatitis C virus antibody-positive; 24 cases were hepatitis B surface antigen-positive; 3 cases were positive for both hepatitis B surface antigen and hepatitis C virus, and 41 cases were negative for both. Ninety-nine of the 157 cases were diagnosed with chronic hepatitis (CH), and 58 cases had liver cirrhosis (LC) based on histological examination. Pathological diagnosis was performed according to General Rules for the Clinical and Pathological Study of Primary Liver Cancer edited by Liver Cancer Study Group of Japan [17]. In this study, gross type was evaluated as either simple nodular type (SN type) or non-SN type (e.g. small nodular type with indistinct margin, simple nodular type with extranodular growth, confluent multinodular type, and infiltrative type). The highest histological grade in the tumor was regarded as its histological grade.

All tissues were immunohistochemically examined for DCP and NX-DCP expressions using anti-DCP antibody (MU-3, 1:500 dilution; EIDIA, Tokyo, Japan), anti-NX-DCP antibody (P-16, 1:1000 dilution; EIDIA, Tokyo, Japan), and BenchMarkXT (Ventana Automated Systems, Inc, Tucson, AZ). All slides were evaluated by two of the authors (A.S and J.A). DCP and NX-DCP expressions were evaluated according to staining intensity and stained area (0–1) within the tumor. The staining intensity was graded as 0, negative; 1, weakly positive; 2, moderately positive; or 3, strongly positive. The expression score was calculated by multiplying staining intensity grade by percentage of stained area. In this analysis, the expression scores of DCP and NX-DCP were categorized as either negative, low expression, or high expression. The median value of expression scores was used to separate the low and high expression groups in tissue positive cases. The relationship between the expression scores of DCP or NX-DCP, and clinicopathological features of HCC, such as gross type, tumor size, histological grade, growth type, presence or absence of capsule formation, capsule infiltration, intrahepatic metastasis (im) and portal vein invasion (vp), was analyzed.

Additionally, serum DCP and NX-DCP levels were determined in 89 patients, including 4 patients taking warfarin, and the DCP/NX-DCP ratio was calculated by dividing serum DCP by serum NX-DCP. Forty mAU/mL, 90 mAU/mL, and 1.5 were used as cutoff levels of DCP, NX-DCP, and DCP/NX-DCP ratio, respectively [10, 12, 14–16]. The relationship between clinicopathological features and serum DCP level, NX-DCP level, or DCP/NX-DCP ratio was also analyzed.

Non-cancerous liver tissues as well as tissue samples obtained from 15 patients with obstructive jaundice who underwent surgical resection of liver due to bile duct cancer or intrahepatic biliary stone were immunohistochemically examined for DCP and NX-DCP expression. Immunostaining of non-cancerous tissues was evaluated into 4 grades according to the stained area; i.e., negative, slightly positive, moderately positive, and strongly positive.

The expression scores of DCP and NX-DCP, serum levels of DCP and NX-DCP, and DCP/NX-DCP ratio were compared with clinicopathological features using χ^2^ or Fisher’s exact test. The relationship between expression score and serum DCP level, NX-DCP level, or DCP/NX-DCP ratio was examined using Spearman’s correlation. The survival rates were calculated using Kaplan Meier method, and differences were evaluated by the Wilcoxon test. Differences were considered significant at p < 0.05.

This study was approved by the Ethics Committee of Kurume University [approval #11212]. Written informed consent was obtained from cases from 2009 to 2012 prior to participation. Our institutional review board waived the need for written informed consent from cases of 2007 and 2008 because the data for these patients were retrospectively analysed.

## Results

### Expression of DCP and NX-DCP in HCC tissue

**Fig 1:**
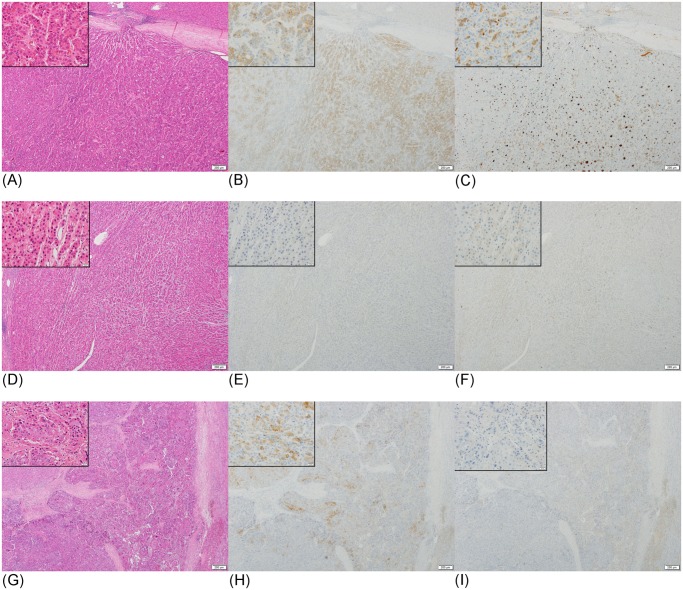
Immunostaining of DCP and NX-DCP in well, moderately, and poor differentiated HCCs. (A-C) A case of moderately differentiated HCC showing both DCP and NX-DCP expression. HE stain (A), and immunostain for DCP (B) and NX-DCP (C). DCP-positive area overlaps with NX-DCP-positive area. (D-F) A case of well-differentiated HCC showing NX-DCP expression, but no DCP expression. HE stain (D), and immunostain for DCP (E) and NX-DCP (F). (G-I) A case of poorly differentiated HCC showing DCP expression, but no NX-DCP expression. HE stain (G), and immunostain for DCP (H) and NX-DCP (I).

DCP and NX-DCP expressions were found to be positive in 83 of 157 cases (53%) and 101 of 157 cases (64%), respectively, in HCC tissues. Fifty-five cases were positive for both DCP and NX-DCP (Fig. 1A-C), and 28 cases were negative for both. DCP-positive area partly or entirely overlapped with NX-DCP-positive area in 51 of the 55 cases (Fig. 1A-C). Forty-six cases were positive for NX-DCP but negative for DCP (Fig. 1D-F), and 28 cases were positive for DCP only (Fig. 1G-I).

**Fig 2:**
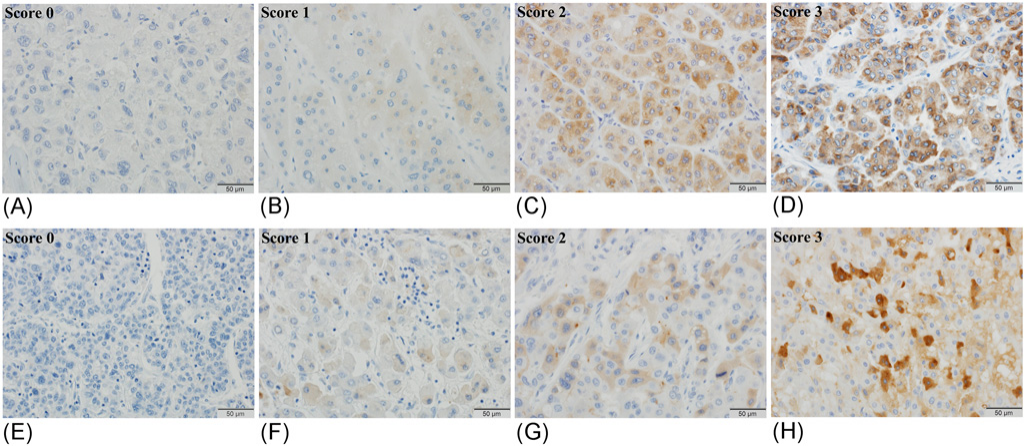
Immunostaining of DCP (A-D) and NX-DCP (E-H) in HCC tissues. The staining intensity was graded into 4 levels, i.e., 0, negative; 1, weakly positive; 2, moderately positive; 3, strongly positive.

We calculated expression scores of DCP and NX-DCP in HCC tissues according to the method described above. A typical immunostaining photomicrograph of each grade is shown in Fig. 2. The expression scores of DCP and NX-DCP were 0.54 ± 0.4 and 0.15 ± 0.02 (mean ± SD), respectively. In the positive cases, the median DCP and NX-DCP expression scores were 0.8 and 0.06, respectively. Table 1 shows the relationship between expression score and clinicopathological features. High DCP expression was significantly more frequent in cases with intrahepatic metastasis (im)(p < 0.05) and tended to be more frequent in portal vein invasion (vp)(p < 0.09), than those with low DCP expression. On the other hand, the cases with high NX-DCP expression showed a significantly lower histological grade, and less frequent im or vp (p < 0.05) than those with low NX-DCP expression score. There were no significant differences in other clinicopathological features, such as growth type, capsule formation, and capsule invasion between low and high expression scores of DCP or NX-DCP. Microscopically, tumor casts in portal vein were observed in 19 of the 157 sections, and 12 of the 19 cases showed DCP expression.

**Table 1:** Relationship between clinicopathological features and DCP or NX-DCP expression in HCC tissue.

Clinicopathological findings		DCP expression score (n)				NX-DCP expression score (n)			
		0 (74)	0–0.8 (43)	0.8< (40)	p value	0 (56)	0–0.06 (62)	0.06< (39)	p value
Sex (%)	Male	74.3	83.7	65.0	0.23	75.0	77.4	69.2	0.65
	Female	25.7	16.3	35.0		25.0	22.6	30.8	
Viral infection (%)	HBsAg positive	10.8	25.6	12.5	0.28	14.3	17.7	12.8	0.74
	HCVAb positive	59.5	60.5	47.5		62.5	53.2	53.8	
	HBsAg & HCVAb positive	2.7	0	2.5		1.8	3.2	0	
	Negative	27.0	14.0	37.5		21.4	25.8	33.3	
Background liver (%)	Chronic hepatitis	66.2	51.2	70.0	0.17	66.1	62.9	59.0	0.78
	Liver cirrhosis	33.8	48.8	30.0		33.9	37.1	41.0	
Tumor size (%)	<20mm	32.4	16.3	30.0	0.15	26.8	33.9	17.9	0.32
	≥20mm	67.6	83.7	70.0		73.2	66.1	82.1	
Gross type (%)	SN ^†^	62.2	58.1	45.0	0.21	48.2	66.1	53.8	0.13
	Non-SN	37.8	41.9	55.0		51.8	33.9	46.2	
Histological grade (%)	Well diff^‡^.	12.2	9.3	2.5	0.34	1.8	8.1	20.5	0.02*
	Moderately and Poorly diff.	87.8	90.7	97.5		98.2	91.9	79.5	
Capsule formation (%)	Absent	29.7	37.2	47.5	0.17	39.3	32.3	38.5	0.69
	Present	70.3	62.8	52.5		60.7	67.7	61.5	
Capsule invasion (%)	Absent	33.8	41.9	47.5	0.34	41.1	35.5	43.6	0.69
	Present	66.2	58.1	52.5		58.9	64.5	56.4	
Intrahepatic metastasis (%)	Absent	94.6	74.4	82.5	0.02†	73.2	90.3	97.4	0.01†
	Present	5.4	25.6	17.5		26.8	9.7	2.6	
Portal vein invasion (%)	Absent	56.8	37.2	42.5	0.09	33.9	56.5	53.8	0.03‡
	Present	43.2	62.8	57.5		66.1	43.5	46.2	

^†^SN; simple nodular type,

^‡^diff; differentiation.

* p = 0.02, vs. Moderately and Poorly diff.

† p = 0.01–0.02, vs. Present.

‡ p = 0.03, vs. Present.

**Fig 3:**
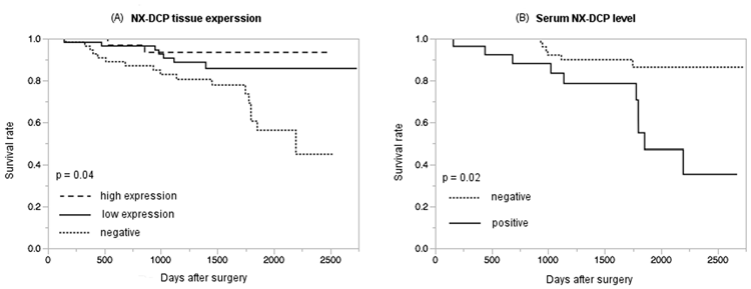
Survival rate of HCC patients according to NX-DCP expression level in HCC tissue or serum NX-DCP level. (A) The cases with negative NX-DCP expression (dotted line) showed lower survival rate (p = 0.04). Dashed line, high NX-DCP expression; solid line, low NX-DCP expression; dotted line, negative cases. (B) The survival rate of the serum NX-DCP positive cases (solid line) was significantly lower than that of negative cases. Dotted line, serum NX-DCP negative; solid line, positive cases.

Survival rate was significantly lower in the NX-DCP expression-negative group than in the low or high NX-DCP expression groups (p = 0.04) (Fig. 3A). Overall survival was lowest in the NX-DCP expression-negative group. There was, however, no significant difference in survival between DCP expression-negative cases, and those with the low or high DCP expression scores (p = 0.43).

### Expression of DCP and NX-DCP in non-cancerous liver tissue

**Fig 4:**
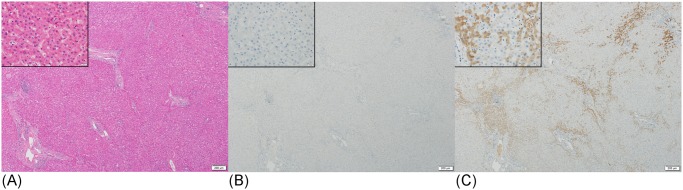
Immunostaining of DCP and NX-DCP in non-cancerous liver tissue. (A-C) Non-cancerous liver tissue showing scattered areas with strong NX-DCP expression, but no areas of DCP expression. HE stain (A), and immunostain for DCP (B) and NX-DCP (C).

**Fig 5:**
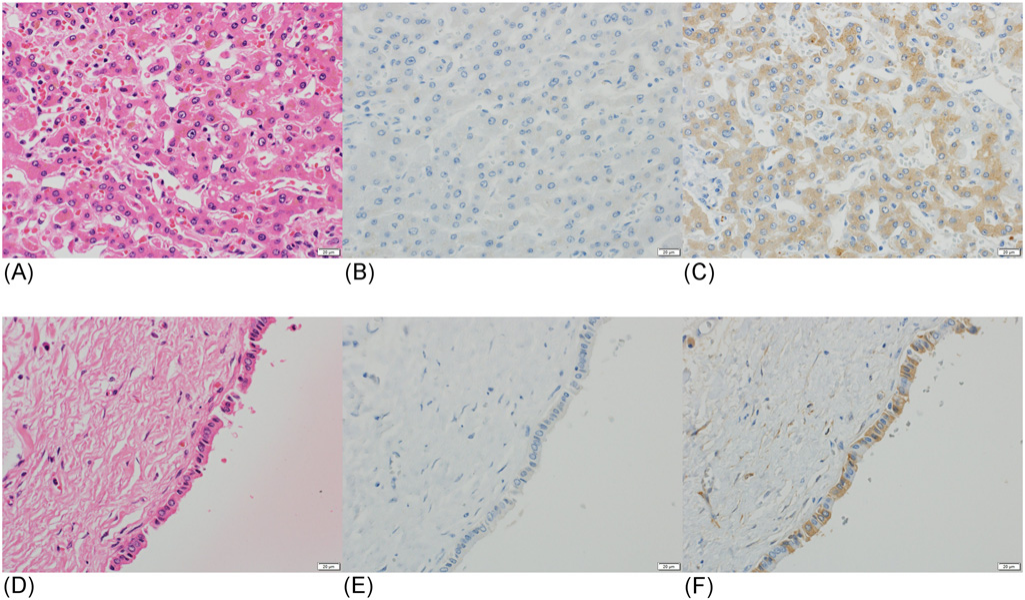
Immunostaining of DCP and NX-DCP in liver tissue of a patient with obstructive jaundice. (A-C) Liver tissue in a case of obstructive jaundice showing NX-DCP expression, but no DCP expression. HE stain (A), and immunostain for DCP (B) and NX-DCP (C). (D-F) Biliary epithelial cells of the same case showing NX-DCP expression, but no DCP expression. HE stain (D), and immunostain for DCP (E) and NX-DCP (F).

DCP expression in non-cancerous tissue was observed in only one case of obstructive jaundice. On the other hand, NX-DCP expression in non-cancerous tissue was detected in 115 of 141 (82%) HCC cases not taking warfarin (Fig. 4A-C) and in all 6 cases taking warfarin. Moreover, 55 of the 141 (39%) cases not taking warfarin and 4 of the 6 (67%) cases taking warfarin showed moderately to strongly positive NX-DCP-stained areas in non-cancerous tissues. In most of these cases non-cancerous tissue adjacent to HCC also expressed NX-DCP. The stained area was more extensive in the cases taking warfarin than in those not taking warfarin. There was no correlation between fibrosis and the expression of NX-DCP in non-cancerous tissue. All 15 cases of obstructive jaundice had NX-DCP expression, and 9 of the 15 (60%) cases showed moderately to strongly positive NX-DCP-stained areas (Fig. 5A-C). Nine of the 15 (60%) cases with obstructive jaundice showed NX-DCP expression in the biliary epithelium (Fig. 5D-F).

### Correlations between serum DCP level and tissue DCP expression, and between serum NX-DCP level and tissue NX-DCP expression in HCC patients

There was a significant correlation between serum DCP level and DCP expression score in HCC patients (rs = 0.479, p < 0.01). However, no significant correlation was observed between serum NX-DCP level and NX-DCP expression score (rs = 0.151, p = 0.08). There was no significant difference in serum NX-DCP level between the cases with CH and LC when the cases taking warfarin were excluded.

Serum DCP was positive (≥ 40 mAU/L) in 50 of 89 cases (56%), and serum NX-DCP was positive (≥ 90 mAU/L) in 28 cases (31%). DCP/NX-DCP ratio was positive (≥ 1.5) in 39 of 89 cases (44%), and DCP/NX-DCP ratio of 4 cases taking warfarin ranged from 0.91 to 2.13. The relationship between clinicopathological features and serum DCP level, serum NX-DCP level, or DCP/NX-DCP ratio is shown in Table 2. Large (≥ 20 mm) tumors and vp were significantly more frequent in serum DCP-positive, serum NX-DCP-positive, or DCP/NX-DCP ratio-positive cases (p < 0.05 or p < 0.01). Also, moderately and poorly differentiated HCCs were significantly more frequent in serum DCP-positive or DCP/NX-DCP ratio-positive cases (p < 0.05). The survival rate of the serum NX-DCP-positive cases was significantly lower than that of negative cases (p = 0.02) (Fig. 3B). The serum DCP-positive or DCP/NX-DCP ratio-positive cases tended to show poor prognosis (DCP-positive, p = 0.33 and DCP/NX-DCP ratio-positive, p = 0.66).

**Table 2:** Relationship between clinicopathological features and serum DCP level, serum NX-DCP level or DCP/NX-DCP ratio in HCC.

Clinicopathological findings		Serum DCP level (n)			Serum NX-DCP level (n)			DCP/NX-DCP ratio (n)		
		40> (39)	≥40 (50)	p value	90> (61)	≥90 (28)	p value	1.5> (50)	≥1.5 (39)	p value
Sex (%)	Male	66.7	78.0	0.23	67.2	85.7	0.12	68.0	79.5	0.33
	Female	33.3	22.0		32.8	14.3		32.0	20.5	
Viral infection (%)	HBsAg positive	17.9	20.0	0.93	23.0	10.7	0.49	14.0	25.6	0.57
	HCVAb positive	66.7	60.0		62.3	64.3		70.0	53.8	
	HBsAg & HCVAb positive	2.6	2.0		3.3	0		2.0	2.6	
	Negative	12.8	18.0		11.5	25.0		14.0	17.9	
Background liver (%)	Chronic hepatitis	53.8	68.0	0.17	57.4	71.4	0.30	56.0	69.2	0.20
	Liver cirrhosis	46.2	32.0		42.6	28.6		44.0	30.8	
Tumor size (%)	<20mm	46.2	22.0	0.02*	41.0	14.3	0.02*	44.0	17.9	0.02*
	≥20mm	53.8	78.0		59.0	85.7		56.0	82.1	
Gross type (%)	SN ^†^	64.1	60.0	0.69	63.9	57.1	0.05	66.0	56.4	0.36
	Non-SN	35.9	40.0		36.1	42.9		34.0	43.6	
Histological grade	Well diff^‡^.	15.4	0	<0.01†	9.8	0	0.21	12.0	0	<0.01†
	Moderately and Poorly diff.	84.6	100		90.2	100		88.0	100	
Capsule formation (%)	Absent	35.9	32.0	0.70	37.7	25	0.40	34.0	33.3	0.94
	Present	64.1	68.0		62.3	75		66.0	66.7	
Capsule invasion (%)	Absent	41.0	34.0	0.50	41.0	28.6	0.37	38.0	35.9	0.84
	Present	59.0	66.0		59.0	71.4		62.0	64.1	
Intrahepatic metastasis (%)	Absent	89.7	80.0	0.34	88.5	75.0	0.19	88.0	79.5	0.42
	Present	10.3	20.0		11.5	25		12.0	20.5	
Portal vein invasion (%)	Absent	59.0	32.0	0.01‡	52.5	25	0.03‡	60.0	23.1	<0.01‡
	Present	41.0	68.0		47.5	75		40.0	76.9	

^††^SN; simple nodular type,

^‡^diff; differentiation.

* p = 0.02, vs. ≥20mm.

† p < 0.01, vs. Moderately and Poorly diff.

‡ p < 0.01–0.03, vs. Present.

## Discussion

Previous studies on the relationship between tissue DCP expression, serum DCP level, and clinicopathological findings of HCC [18–21] reported that 50–75.7% cases showed DCP expression in HCC cells immunohistochemically. In the present study, DCP expression was observed in 53% of the HCC cases. The differences in positive rate might be due to the use of different immunohistochemical stain kits and/or criteria for positive expression. In addition, the previous studies [18–21] and present study revealed that tissue DCP expression is closely associated with malignant properties of HCC (e.g., non-SN type, high histological grade, and im). As to gross classification of HCC, previous studies revealed that non-SN type HCC was a statistically significant risk factor for microscopic vascular invasion, tumor recurrence, and disease-specific death [5, 22, 23]. We can speculate on the reasons for this close relationship based on the data reported previously. Murata et al. reported that cytoskeletal changes during epithelial mesenchymal transition (EMT) induced by chemicals or hypoxia played a critical role in DCP production via impairment in vitamin K uptake [24, 25]. EMT is widely known as an initial step of tumor progression in various carcinomas [26]. Tumor cells that acquired mesenchymal features are best equipped to intravasate. Another possible mechanism that explains the relationship is the proliferative effects of DCP on endothelial cells and HCC cells [27].

In the previous studies serum DCP level did not always correlate with DCP expression in HCC tissue [18, 21]. However, they evaluated this correlation using the percentage of stained area only. We found that DCP expression in HCC tissue shows a close correlation with serum DCP level when the expression is evaluated using staining intensity in addition to percentage of stained area.

Several studies reported that serum NX-DCP was useful for detection of HCC. Recently, Takeji et al. reported that high serum NX-DCP level was significantly associated with worse prognosis for patients with high stage HCC. They speculated NX-DCP levels reflected hepatic functional reserve of non-cancerous liver tissue of HCC patients [12]. Tanaka et al. [16] found that the serum levels of NX-DCP in the patients with vascular invasion were significantly higher than those without vascular invasion and that the overall survival of the NX-DCP positive group was significantly lower than that of the NX-DCP negative group in stage IV. These results may indicate that serum NX-DCP is associated with malignant behaviors of HCC patients. Regarding NX-DCP expression in HCC tissues, they found very faint expression in HCC tissues. In the present study, we confirmed NX-DCP expression in HCC tissue in 64% of the cases, but NX-DCP expression score was relatively low and averaged less than 1/3 that of DCP. We found that the cases with high NX-DCP expression scores had significantly lower histological grade, and less frequent im or vp. We also found that serum NX-DCP-positive cases had significantly larger tumor size, more frequent vp, and a worse prognosis, and that the serum NX-DCP level did not correlate with NX-DCP expression in HCC tissues. It is not clear why tissue NX-DCP expression did not correlate with serum NX-DCP level. There are no previous studies focused on this issue. We clarified that (1) NX-DCP expression is more frequent and extensive than DCP expression in non-cancerous tissue; and (2) expression score of NX-DCP in HCC tissues was lower than that of DCP. Based on these results, we surmise that most serum NX-DCP might be from non-cancerous tissue. We need further study to confirm this speculation.

DCP expression was observed in 11.8–25.7% of cases in non-cancerous tissue adjacent to HCC [18, 19]. Inagaki et al. [19] reported that DCP expression in non-cancerous liver was significantly correlated with malignant properties of HCC. On the other hand, Tanaka et al. [16] reported that DCP expression was not found in non-cancerous tissue. Indeed, in our study, DCP expression in non-cancerous tissue was found in only one case with obstructive jaundice. The reason for the differences among these results is not clear, but may have to do with differences in immunohistochemical techniques and conditions, and different interpretation criteria.

NX-DCP expression in non-cancerous liver tissue was found in 82% of cases in the present study. Tanaka et al. [16] reported strong NX-DCP expression in non-cancerous liver tissue in all cases, including warfarin users. In our study, all cases of obstructive jaundice and all warfarin users showed NX-DCP expression in non-cancerous tissue to various degrees. These findings are consistent with previous studies showing that NX-DCP is a specific marker of vitamin K deficiency under physiological conditions. Interestingly, we are the first to report finding NX-DCP expression in the biliary epithelium in 9 of 15 patients (60%) with obstructive jaundice. It is not clear why biliary epithelium were positive for NX-DCP only under the condition of obstructive jaundice. Further studies using a large number of patients should be conducted in order to clarify this mechanism.

Many studies have shown that the DCP/NX-DCP ratio is useful to detect HCC in warfarin-taking patients with positive serum DCP level [10, 13]. We found that DCP/NX-DCP ratio could also be an indicator of tumor progression. It is still controversial which cutoff values should be used for accurate diagnosis. Some previous reports used 1.5 [2, 10] and a recent one used 1.0 [13]. In the present study, 3 of 4 HCC cases taking warfarin had a DCP/NX-DCP ratio of less than 1.5. In order to establish an accurate cutoff value, a further large-scale study including various types of patients (e.g., HCC patients taking warfarin, obstructive jaundice patients, and so on) should be conducted.

Alpha fetoprotein (AFP) and AFP-L3 also have served as a tumor marker of HCC, and these have been reported to be useful in early detection of HCC [28]. However, there are no previous studies that AFP (AFP-L3) is useful for detecting biological properties of HCC, such as vp and im. In the present study, we found that not only serum DCP and NX-DCP levels, but also tissue DCP and NX-DCP expressions were useful for evaluation of biological properties of HCC.

## Conclusion

Two different types of abnormal prothrombin, DCP and NX-DCP, were produced in HCC tissues, but had different expression levels and exhibited different biological properties. Our data suggest that increases in DCP expression in HCC tissue, serum DCP or NX-DCP level, and DCP/NX-DCP ratio were closely related with malignant properties of HCC.
